# Prevalence of depression and its correlates among undergraduate health science students in Mogadishu, Somalia: a cross-sectional study

**DOI:** 10.1186/s12888-025-06553-5

**Published:** 2025-02-01

**Authors:** Bashiru Garba, Samiro Ali Mohamed, Maria Mowlid Mohamed, Hodo Aideed Asowe, Najib Isse Dirie, Yushau Umar, Jamal Hassan Mohamoud, Mohamed Hussein Adam, Jihaan Hassan, Fartun Abdullahi Hassan Orey, Abdullahi Abdirahman Omar, Ibrahim Abdullahi Mohamed, Mohamed Mustaf Ahmed, Samira Abdullahi Moalim

**Affiliations:** 1 Department of Public Health, Faculty of Medicine and Health Sciences, SIMAD University, Mogadishu, Somalia; 2 Department of Nursing and Midwifery, Faculty of Medicine and Health Sciences, SIMAD University, Mogadishu, Somalia; 3 Department of Urology, Dr. Sumait Hospital, Faculty of Medicine and Health Sciences, SIMAD University, Mogadishu, Somalia; 4 National Veterinary Research Institute, Vom 930101, Jos, Plateau State Nigeria; 5 Department of Veterinary Public Health and Preventive Medicine, Faculty of Veterinary Medicine, Usmanu Danfodiyo University, Sokoto, Nigeria; 6 Department of Paediatrics and Child Health, Dr. Sumait Hospital, Faculty of Medicine and Health Sciences, SIMAD University, Mogadishu, Somalia; 7 Dr. Sumait Hospital Affiliate of SIMAD University, Mogadishu, Somalia; 8 Faculty of Medicine and Health Sciences, SIMAD University, Mogadishu, Somalia

**Keywords:** Academic performance, Depression, Emotional distress, Humanitarian crisis, Mogadishu

## Abstract

**Background:**

Depression among undergraduate health science students is increasingly recognized as a significant public health issue globally. These students face immense academic pressure, often leading to stress and mental exhaustion, particularly for students studying in a humanitarian crisis situation. However, there is a scarcity of information in this regard in higher education institutions in Somalia. Hence, this investigation was conducted to assess the prevalence of depression and its correlates health science students in Somalia.

**Methods:**

A cross-sectional study was conducted on 321 health science students from some universities in Mogadishu, Somalia. The data was collected from students studying medicine, nursing and midwifery, laboratory science and public health at SIMAD university, Banadir university, Mogadishu university and Jamhuriya university, all located in Mogadishu. Simple random sampling based on class list was used to select the participants and a web-based self-administered Patient Health Questionnaire (PHQ-9) was used to screen for depression. The collected data were cleaned and checked for completeness before exporting into SPSS version 27 Statistical software for analysis, where descriptive statistics as well as logistic regression analysis was done to determine the independent predictors of the outcome variable. Significant association was determined at a 95% confidence interval and *p*-value < 0.05.

**Result:**

The prevalence of depression among the students was 58.6%. A total of 41.5% (133/321) of the participants were free from depression (Normal), while 30.8% had mild depression, 27.1% had moderate depression and 0.6% had severe depression. Analysis of the association between the sociodemographic variables and depressive state of the participant showed statistical significance (*p*-value = 0.045) for course of study. Students studying nursing and midwifery had the highest case of depression with 68.6% (59/86), followed by public health 66.7% (30/45), laboratory science 52.3% (34/65), and medicine having the least number of depressed students with 52.0% students (65/125). Similarly, gender of students was also found to be statistically significant (*p*-value = 0.001) with female students having 65% depression which is 41.1% of the total students studied.

**Conclusion:**

The prevalence of depression among health science students in Mogadishu was high and positively associated with gender, having trouble with authorities, emotional problems, as well as experience of sexual abuse or violence. Hence, routine screening and monitoring of students’ mental health state on the campus and providing mental health services is necessary to address this growing problem.

**Supplementary Information:**

The online version contains supplementary material available at 10.1186/s12888-025-06553-5.

## Introduction

Depression is a serious mental health disorder attributed to a combination of psychosocial, hereditary and environmental factors [37]. The condition is characterized by persistent feelings of sadness, hopelessness, and a lack of interest or pleasure in routine activities [36]. Recent estimates by the World Health organization suggest that depression is the leading cause of ill health and disability globally, with an estimated prevalence of 5% among adults [44, 46]. Depression affects people of all ages and backgrounds, with varying degrees of severity, from mild to debilitating forms that impact daily functioning. For instance, depression in health science students often stems from academic pressure, burnout, and fear of failure, which often impact on their ability to study and empathize with patients. In the general public on the other hand, it may arise from socioeconomic factors, life events, or chronic stress, affecting daily functioning and interpersonal relationships, with varied access to coping resources [27, 42]. In a related development, the WHO has identified strong links between depression and other noncommunicable disorders and diseases including substance use disorders, diabetes and cardiovascular disease [44]. On the flip side, depressed individuals also have an increased risk of developing certain chronic illnesses such as heart disease, stroke, and other neurological diseases like Alzheimer’s among others [19].

From a public health perspective, the economic impact of depression is profound, including direct costs related to healthcare services and indirect costs due to lost productivity and absenteeism [12]. The social stigma surrounding mental health issues characterized by discrimination, prejudice, and negative stereotypes can also deter individuals from seeking timely care, thereby resulting in hindered health outcomes [4]. Depression is a pervasive and debilitating mental health issue, particularly in countries experiencing humanitarian crises such as Somalia. Depression in Somalia is a significant mental health challenge, influenced by decades of conflict, displacement, poverty, and limited healthcare access [39]. Cultural practices often prioritize spiritual remedies over medical interventions, necessitating awareness campaigns, mental health services expansion, and integration of culturally sensitive approaches. Prolonged conflict, political instability, and recurrent natural disasters including five consecutive seasons of no rain have severely impacted the mental well-being of the Somali population [20, 28]. The persistent threat of violence, displacement, loss of livelihoods, and economic hardship tend to exacerbate the prevalence of depression [26, 45].

Health science students are among the high-risk group for depression owing to the intense academic pressures, demanding clinical responsibilities, and emotional strain from personal stressors [3, 33]. Many studies indicate an exceptionally high prevalence of depression and anxiety among medical students compared to the general student population [1, 41]. Many of the contributing factors are either personal or institutional. In case of conflict affected countries, the Learning environment, educational debt, hard workload, and sleep disturbances are some of the most common risk factors that predispose health science students to this public health challenge [1].

In light of a recent report of 33.5% depression among adults living with HIV/AIDS currently undergoing anti-retroviral therapy in Mogadishu [30], tracking this important public health problem can provide useful insights for clinicians and policy makers regarding the status of depression among Somali youths. In addition, highlighting the sociodemographic, behavioural, and societal costs and consequences of depression can guide decisions aimed at improving the lives of students battling with depression. With this in mind, this investigation sought to determine the prevalence of depression and its associated factors among undergraduate students studying medicine, public health, laboratory sciences, as well as nursing and midwifery science in Somalia.

## Methods

### Study design

An institution-based quantitative cross-sectional study was undertaken among 321 undergraduate students studying medicine, public health, medical laboratory sciences, nursing and midwifery science at both public and private universities in Mogadishu, Somalia.

### Study setting and context

The study was conducted in four different public and private universities in Mogadishu, Banadir region, Somalia. The programs included are the health science courses including Doctor of Medicine MBBS which is a seven-year program including the mandatory foundation year, while the Nursing and Midwifery, Public Health, and Laboratory Sciences programs are often complete in 5 academic years including the foundation year.

Medical and health education in Somalia has faced significant challenges due to decades of conflict, instability, and limited resources [32, 38]. However, in recent years, the country has made significant strides in rebuilding its health sector through educational institutions like SIMAD University, Banadir University, Jamhuriyya University, and Mogadishu University among many other which offer health sciences training programs. Although, the curriculum often lacks standardization, and there are shortages of qualified faculty and modern training facilities. Notwithstanding, partnerships with international organizations, members of the diaspora community, and other local initiatives are helping to address these gaps.

The present study was conducted in the period between January 2024 and June 2024 to assess the prevalence of depressive symptoms among these undergraduate health science students.

### Study population and selection criteria

A multistage stratified sampling technique was used to target a total of 326 health science students from four departments in each of the four selected universities in the study area. Each respondent was selected by simple random sampling technique using the class register as a frame. In the event a student refused to participate, the next student was contacted. Undergraduate students pursuing a Bachelor of Medicine and Bachelor of Surgery, Public Health, Laboratory Science, as well as Nursing and Midwifery Sciences constituted the study population. The 7-year MBBS curriculum consists of 1 year of mandatory foundation studies recommended by the Somali National Government, followed by 2 years of preclinical training in basic biomedical sciences and 3 years of clinical rotations. On the other hand, the Public Health, Laboratory Science, and Nursing and Midwifery Sciences program is completed in 5 years comprising of 1 foundation year, 2 years of preclinical and 2 years of clinical training.

### Sample size estimation

The sample size was calculated using the Cochrane single population proportion formula; Cochran formula: n = (Z^2 * p * (1-p)) / e^2. The parameters considered were n = sample size, Z = Z-score (1.96 for a 95% confidence level), p = proportion of depression among undergraduate students as previously reported (28.2%) [5], d = desired precision or margin of error (5%). In addition, a 5% non-response rate was added to get a total sample size of 326.

Samples were allocated to each of the four universities proportionately based on the total number of students in the university register as well as across each department. Accordingly, 40, 68, 118, and 100 students were allocated to SIMAD university, Jamhuriya university, Banadir university, and Mogadishu university respectively.

### Data-collection tools and procedure

A self-administered, well-structured questionnaire was developed based on an extensive review of the literature to collect data from participants. Data were collected using a web-based questionnaire designed with Google Forms, ensuring accessibility and convenience (Supplementary 1).

Class representatives, serving as class leaders, facilitated dissemination of the survey link through class WhatsApp groups and assisted in identifying selected participants using class registers under the guidance of the research team. Their role was limited to logistical support and closely monitored to reduce bias.

The questionnaire comprised three sections. The first section collected sociodemographic characteristics, the second included ten “Yes” or “No” questions assessing potential risk factors associated with depression, and the third featured nine 4-point Likert scale items from the Patient Health Questionnaire-9 (PHQ-9). The PHQ-9, adapted from validated tools in previous studies [6, 35]. The PHQ-9 is a widely recognized instrument for screening, diagnosing, and assessing the severity of depression [17]. To further ascertain the reliability and internal consistency of the questionnaire, Cronbach’s alpha Coefficient was calculated and found to have a good overall score of 0.86. Each item in the PHQ-9 is scored from 0 to 3, corresponding to “not at all,” “several days,” “more than half the days,” and “nearly every day,” respectively. The total score ranges from 0 to 27, with higher scores indicating greater severity of depressive symptoms.

### Measurement of depression

A diagnosis of depressive status of a participant was made based on the following PHQ9 grading; 0–4, 5–9, 10–14, 15–19 and 20–27, which correspond to no depression, mild depression, moderate depression, and severe depression, respectively [22].

### Data quality management

Validity and completeness of data was assured via regular supervision to ascertain consistency on daily basis. In the event an incomplete questionnaire was detected, the data collection supervisor removed the entry as it was difficult to trace the specific participant.

### Data management and analysis

The web-based data submissions from Google forms were downloaded in Microsoft Excel format before cleaning and allocation of codes (Supplementary 2). The formal analysis was performed using SPSS Statistical Software v27 (IBM SPSS). Descriptive analyses were in the form of frequencies and percentages was used for each of the categorical variables, while the age of the respondents was expressed as mean ± SD. To understand the associations between depression and the potential risks factors and participants demographics, Chi-square (χ2) tests and logistic regressions were performed. A *P*-value of < 0.05 was considered statistically significant.

All variables from socio-demographic characteristics of participants and the potential risk factors in the bivariate analysis were fitted into the binary logistic regression model to identify independent predictors of depression among the students.

## Results

### Participants demographics

A total of 321 participants with a median age of 20 (Mean ± SD-20.5 ± 2.4) years responded to the study representing a response rate 98.5%. Although the age ranged from 15–40 years, 96% of the students are aged 15–25 years (Table 1). The majority of the participants were female (62.9%), while only 5% were married with the majority (95%) being single (Supplementary 3).

**Table 1:** Socio-demographic characteristics of the participants

Variable	Frequency	(%)
**Age (years)**		
15–25 years	308	95.9
26–30 years	8	2.5
31–40 years	4	1.3
More than 40 years	1	0.30
**Mean ± SD (20.5 ± 2.4)**		
**Gender Male**		
Female	202	62.9
Male	119	37.1
**Marital status**		
Married	16	5.0
Single	305	95.0
Widowed/Divorced	0	0.0
**Level of study**		
Clinical	156	48.6
Pre-clinical	165	51.4
**Family size**		
0–5 members	53	16.5
6–10 members	174	54.2
More than 10 members	94	29.3
**Institution**		
Banadir university	99	30.8
Jamhuriya University of Science & Technology	72	22.4
Mogadishu University	89	27.7
SIMAD University	61	19.1
**Degree Program**		
Laboratory Sciences	65	20.2
Medicine and Surgery	125	38.9
Nursing and Midwifery Sciences	86	26.8
Public Health	45	14.1

### Prevalence of depression

**Fig. 1:**
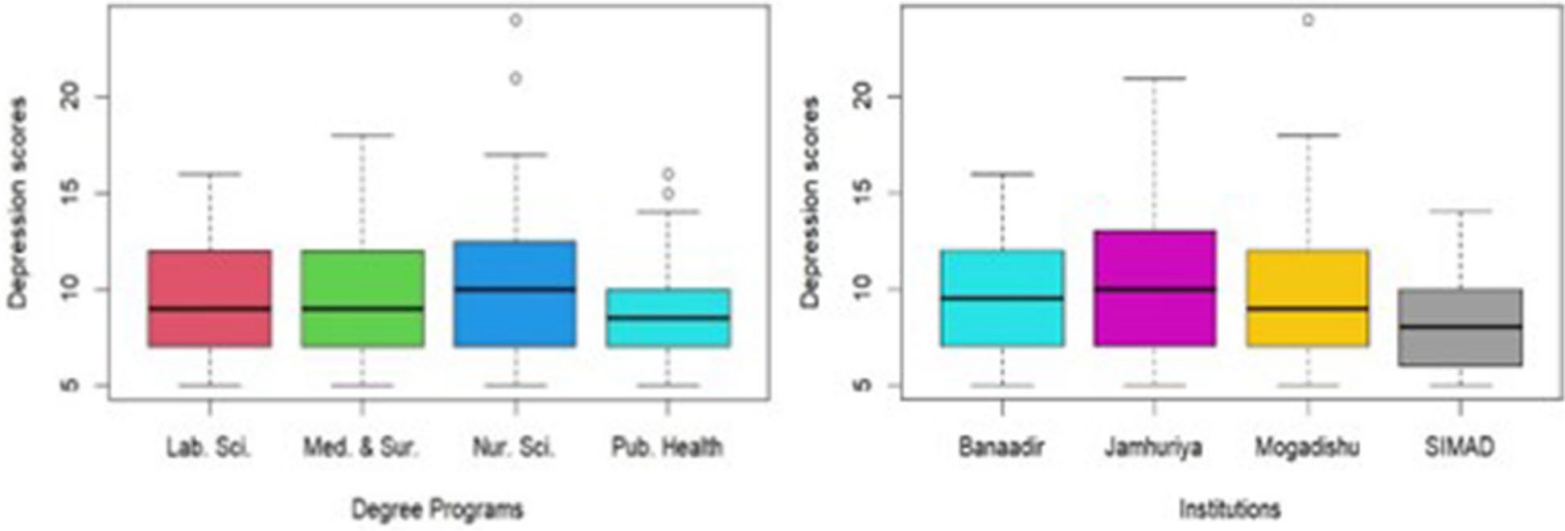
Depression scores according to institution and course of study

**Fig. 2:**
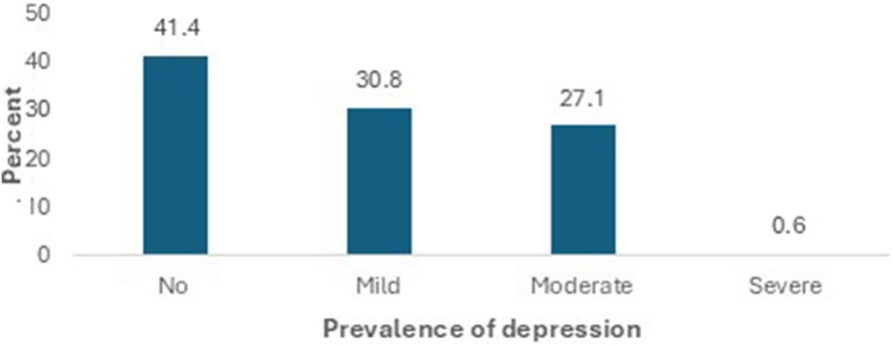
Prevalence of depression among health science students studying in Mogadishu, Somalia

Figures 1 and 2 presents a summary of the scores and prevalence of depression among health science students studying in SIMAD University, Mogadishu University, Banadir University, and Jamhuriya University. Overall, 58.6% (188/321) of the health science students had depression. Prevalence of depression according to institution of study showed that Mogadishu university had the highest rate of depressed students followed by Banadir, Jamhuriya, and SIMAD respectively. On the other hand, depressive score based on course of study revealed that students studying MBBS program had higher score of depression followed by nursing and midwifery students with public health students having the least (Fig. 1).

A total of 41.5% (133/321) of the participants were free from depression (Normal), while 30.8% had mild depression, 27.1% had moderate depression and 0.6% had severe depression (Fig. 2).

Analysis of the association between the sociodemographic variables and depressive state of the participant showed statistical significance (*p*-value = 0.045) for course of study with students studying nursing and midwifery having highest case of depression with 68.6% (59/86), followed by public health 66.7% (30/45), laboratory science 52.3% (34/65), and medicine having the least number of depressed students with 52.0% students (65/125). Similarly, gender of students was also found to be statistically significant (*p*-value = 0.001) with female students having 65% depression which is 41.1% of the total students studied. Table 2 summarizes the association between participants demographic characteristics and depression.

**Table 2:** Demographic variables associated with depression among the study population

**Variable**	**Depression**		**χ 2**	***p*-value**
	**Yes, n (%)**	**No, n (%)**		
**Institution**				
Banadir University	52(52.5)	47(47.5)	4.397	0.222
Jamhuriya University of science and Technology	49(68.1)	23(31.9)		
Mogadishu University	53(59.6)	36(40.5)		
SIMAD University	34(55.7)	27(44.3)		
**Degree Program**				
Laboratory Science	34(52.3)	31(47.7)	8.058	0.045*
Medicine and Surgery	65(52.0)	60(48.0)		
Nursing and Midwifery Science	59(68.6)	27(31.4)		
Public Health	30(66.7)	15(33.3)		
**Age group (years)**				
15—25	182(59.1)	126(40.9)	2.76	0.423
26—30	4(50.0)	4(50.0)		
31—40	1(25.0)	3(75.0)		
Above 40	1(100)	0(00.0)		
**Gender**				
Female	132(65.3)	70(34.7)	10.321	0.001*
Male	56(47.1)	63(52.9)		
**Marita Status**				
Married	10(62.5)	6(37.5)	0.107	0.479
Single	178(58.4)	127(41.6)		
**Level of Study**				
Clinical	94(60.3)	62(39.7)	0.357	0.314
Pre-clinical	94(57.0)	71(43.0)		
**Family size**				
0—5	33(62.3)	20(37.7)	1.249	0.54
6—10	97(55.7)	77(44.3)		
> 10	58(61.7)	36(38.3)		

^*^Statistically significant (bold), χ 2 = Chi square

### Risk factors associated with depression

The logistic regression analysis for independent predictors of depression among the study population showed that, being a male, suffering with emotional problems, experiencing sexual violence or abuse, and having trouble with school authorities were associated with depression (*p* < 0.05).

The result of the adjusted odds of having depression was two-fold higher (OR = 2.07, 95% CI 0.921–4.667), among students studying nursing and midwifery sciences as compared to students studying laboratory science. Additionally, the odds of having depression were approximately four times more likely (OR = 5.053, 95% CI 2.787–9.167) in the students who had emotional problems than their counterparts who reportedly had no emotional problem (Table 3).

**Table 3:** Logistic regression model for independent predictors of depression among the study population

Variables	Regression	Adjusted Odds Ratio (AOR)	95% CI for AOR		*p*-value
**Degree program**			**Lower**	**Upper**	
Laboratory Sciences^a^		1			
Medicine and Surgery	−0.061	0.941	0.457	1.938	0.869
Nursing & Midwifery Sciences	0.729	2.073	0.921	4.667	0.078
Public Health	0.691	1.996	0.768	5.189	0.156
**Gender**					
Female^a^		1			
Male	−0.661	0.517	0.284	0.939	0.030**
**Emotional Problem**					
No^a^		1			
Yes	1.620	5.053	2.787	9.164	0.000**
**Failed examination**					
No^a^		1			
Yes	0.506	1.659	0.846	3.251	0.141
**Loss of friend or family member**					
No^a^		1			
Yes	0.223	1.250	0.689	2.266	0.462
**Financial challenges**					
No^a^		1			
Yes	0.334	1.396	0.777	2.510	0.264
**Chronic Illness/surgery**					
No^a^		1			
Yes	−0.096	0.908	0.318	2.595	0.858
**Physical abuse or trauma**					
No^a^		1			
Yes	0.992	2.697	0.806	9.030	0.108
**Sexual abuse/violence**					
No^a^		1			
Yes	−1.766	0.171	0.039	0.757	0.020**
**Substance abuse/addiction**					
No^a^		1			
Yes	0.052	1.054	0.314	3.536	0.932
**Unplanned pregnancy**					
No^a^		1			
Yes	−0.131	1.099	0.277	2.775	0.823
**Trouble with school authorities**					
No^a^		1			
Yes	1.299	3.666	1.760	7.634	0.000**
Constant	−0.991				

^**^Statistical significance

^a^Reference category

Other notable results based on the odds ration were that students who had trouble with school authorities and suffered physical abuse or trauma were nearly four times (OR = 3.666, 95%CI 1.760–7.634) and almost three times (OR = 2.697, 95%CI 0.806–7.030) more likely to develop depression than students who had no such events. Further, despite not been statistically significant, students who reported failing an examination, loss a friend or unplanned pregnancy were more likely to report depression than their counterparts (Table 3).

## Discussion

Depression is a significant mental health concern that affects individuals across various demographics. However, undergraduate students, particularly those undergoing health science training, have been found to be at a higher risk [16]. This elevated risk is often attributed to the unique pressures associated with medical education, including academic workload, clinical responsibilities, and the emotional toll of patient care. As a consequence, depression is reported to inversely correlate with empathy as well as impede empathic communication among medical students [43].

In this study, we sought to investigate the prevalence of depression and other potential risk factors associated with the condition among health science students at some selected public and private universities in Mogadishu, Somalia, and found an overall high prevalence of 58.6%, in line with previous studies on medical students in India (50.0%) but lower than the 82.6% reported in a scoping review on health science students [3, 15]. Notwithstanding, this figure is largely higher than the prevalence rates reported across different studies in a similar study population. For instance, the finding was higher than the studies carried out in neighbouring Addis Ababa 27.7%, Uganda 30.2%, Kenya 29.2%, Malaysia 36.4%, China 36%, and Benin 39.1% [6, 18, 21, 25, 33, 40]. Conflicts, whether interpersonal, intrapersonal, or societal, are significant stressors that can contribute to the development and exacerbation of depression leading to negative emotions such as frustration, anxiety, anger, and hopelessness. Unfortunately, when conflicts persist or remain unresolved, they can have a profound psychological impact, potentially leading to or worsening depressive symptoms. This is demonstrated in the results of a similar study conducted in Syria where the ongoing conflict was reported to be an additional stressor for undergraduate students studying medicine and related health science courses potentially contributing to poor mental health outcomes [7]. Similarly, a study conducted among residents of Dessie City, Ethiopia who have experienced prolonged armed conflict show that post-traumatic stress disorders, perceived life threat, low social support which are all attributes of conflict affected population have all been found to be significantly associated with depression [10].

This study also revealed that females had higher mean PHQ-9 score compared to their male counterparts, and this was significant. This finding is similar to several studies that found higher depression rates among females compared to males [11, 24]. This disparity maybe influenced by a complex interplay of biological, psychological, social, and academic factors. Previous studies have shown that increased risk of depression among female students can be attributed to their increased sensitivity to interpersonal relationships, as well as hormonal changes [8]. Among the other demographics that were found to be significant is the program of study where students studying MBBS had a higher depression, followed by nursing and midwifery students compared to students from the public health and laboratory science courses. The intense nature of health science education, combined with the unique stressors such as intensive curriculum faced in healthcare training, contributes to this heightened risk. Consistent findings were reported from the study done in a public university located in Riyadh, Saudi Arabia [9].

In this study, all the potential risk factors evaluated were found to significantly contribute to depressive state of the students. These include failing examination, emotional problem, losing a friend or family member, financial challenges, physical and sexual abuse, or suffering from chronic illness, which have all been shown to be closely related to the development and exacerbation of depression. Academic failure, particularly for health science students who place high value on their academic performance, can have a profound impact on self-esteem and self-worth and may lead to feelings of inadequacy, self-doubt, and shame, which can contribute to depressive thoughts [2]. Similarly, ongoing emotional struggles, such as unresolved conflicts in personal relationships, unaddressed physical trauma or sexual abuse, or persistent feelings of loneliness, can act as chronic stressors [31]. It is not uncommon for students to come with a varied and challenging background including experiences of physical or sexual violence, financial difficulties, or substance abuse habits which can make them susceptible to developing anxiety and depression once academic pressure is mounted. Therefore, need to identify these risk factors with a view to instituting plans to tackle them in order for students to study in a conducive learning environment [29].

Logistic depression model for independent predictors of depression among the study participants revealed a significant statistical significance for gender, emotional problem, sexual abuse and violence, as well as having trouble with school authorities. Psychological research reports show a high prevalence of mental health problems among students who have trouble with school authorities as a result of truancy or absenteeism which tend to increase risk of depression and anxiety [14]. Our results showed that worrying after failing an examination, substance abuse or addiction, or unplanned pregnancy were all found to be associated with depression, although not significant. This outcome is similar to other studies that report that increased academic demands and concerns were associated with depression among health science students [33]. In contrast, substance abuse has been found to be associated with major depressive disorder including suicidal ideation [13, 34]. Furthermore, substance abuse and depression among health science students are interconnected issues that pose significant challenges for both individual students and the healthcare profession. This can be attributed to the intense demands of health science education, particularly in countries like Somalia who are in great demand of healthcare workers, coupled with the stress and pressures associated with the training, make health science students particularly vulnerable to mental health issues, including depression. Unfortunately, some students may turn to substance use as a way to cope with these overwhelming stressors, further exacerbating mental health problems [23].

### Study limitations

The PHQ-9 is mostly used for the screening of depression, hence not able to diagnose current major depressive episode. We understand that some student’s (Banadir and Mogadishu university) refusal to participate may be due to concerns about being subjected to stigma, however we didn’t record the number of students that refused, hence the true response rate was not clearly ascertained. Important to also highlight that the participants distribution may not have been proportionate because we compensated participants from institutions that refused with students from the other institutions.

## Conclusion

This study has shown a high prevalence of depression among undergraduate health science students in Mogadishu, Somalia. Also, demonstrate presence of association between depression and some demographics such as gender, course of study, financial challenges, sexual abuse, and having trouble with the school authorities. These findings highlight the need for the Somali educational and health authorities to collaborate and produce robust strategies to address mental health challenges especially among students to prevent new incidences of depression.

## Supplementary Information

Supplementary Material 1.

Supplementary Material 2.

Supplementary Material 3.

## Data Availability

All data generated or analyzed during this study are included in this published article (and its supplementary information files).

## Abbreviations

PHQ-9: Patient Health Questionnaire
SPSS: Statistical Package for Social Sciences
WHO: World Health Organization
HIV/AIDS: Human Immunodeficiency Virus/Acquired Immunodeficiency Syndrome
MBBS: Bachelor of Medicine Bachelor of Surgery
