# Supplementary material for: Prevalence of depression and its correlates among undergraduate health science students in Mogadishu, Somalia: a cross-sectional study
Source: BMC Psychiatry. 2025 Feb 1;25:89. doi: 10.1186/s12888-025-06553-5 (PMC11786374; doi:10.1186/s12888-025-06553-5)
Supplement: Supplementary file 1 — Supplementary Material 1. [file 12888_2025_6553_MOESM1_ESM.docx]

**Questionnaire tool**

**Section 1**

**Sociodemographic characteristics**

1. Institution
   1. Banadir University
   2. [Jamhuriya University of Science and Technology](https://just.edu.so/)
2. Mogadishu University
3. SIMAD University
4. Program Course
   1. Public Health
   2. Laboratory Sciences
   3. Nursing and Midwifery Sciences
   4. Medicine and Surgery
5. Age
   1. 15-25 years
   2. 26-30 years
   3. 31-40 years
   4. More than 40 years
6. Gender
   1. Male
   2. Female
7. Marital status
   1. Married
   2. Single
   3. Widowed
8. Level of study
   1. Pre-clinical
   2. Clinical
9. Family size
   1. 0-5
   2. 6-10
   3. More than 10

**Section 2**

**Exposure to risk factors for depression**

1. Failed an examination.
   1. Yes
   2. No
2. Emotional problems
   1. Yes
   2. No
3. Loss of a friend or family member
   1. Yes
   2. No
4. Financial challenges
   1. Yes
   2. No
5. Chronic illness/surgery
   1. Yes
   2. No
6. Physical abuse or trauma
   1. Yes
   2. No
7. Sexual abuse/violence
   1. Yes
   2. No
8. Substance abuse or addiction
   1. Yes
   2. No
9. Unplanned pregnancy
   1. Yes
   2. No
10. Trouble with school authorities
    1. Yes
    2. No

**Section 3**

**Patient Health Questionnaire-9 (PHQ-9)**

1. Little interest or pleasure in doing things.
   1. Not at all
   2. Several days
   3. More than half the days
   4. Nearly every day
2. Feeling down, depressed, or hopeless.
   1. Not at all
   2. Several days
   3. More than half the days
   4. Nearly every day
3. Trouble falling or staying asleep or sleeping too much.
   1. Not at all
   2. Several days
   3. More than half the days
   4. Nearly every day
4. Feeling tired or having little energy
   1. Not at all
   2. Several days
   3. More than half the days
   4. Nearly every day
5. Poor appetite or overeating
   1. Not at all
   2. Several days
   3. More than half the days
   4. Nearly every day
6. Feeling bad about yourself or that you are a failure or have let yourself or your family down.
   1. Not at all
   2. Several days
   3. More than half the days
   4. Nearly every day
7. Trouble concentrating on things such as reading the newspaper or watching television.
   1. Not at all
   2. Several days
   3. More than half the days
   4. Nearly every day
8. Moving or speaking so slowly that other people could have noticed? or the opposite-being so uneasy or restless that you have been moving around a lot more than usual?
   1. Not at all
   2. Several days
   3. More than half the days
   4. Nearly every day
9. Thoughts that you would be better off dead or hurting yourself in some way.
   1. Not at all
   2. Several days
   3. More than half the days
   4. Nearly every day

**Thank you.**
