# Supplementary material for: Prevalence of depression and its correlates among undergraduate health science students in Mogadishu, Somalia: a cross-sectional study
Source: BMC Psychiatry. 2025 Feb 1;25:89. doi: 10.1186/s12888-025-06553-5 (PMC11786374; doi:10.1186/s12888-025-06553-5)
Supplement: Supplementary file 2 — Supplementary Material 2. [file 12888_2025_6553_MOESM2_ESM.docx]

**CODE BOOK**

**SECTION A: SOCIODEMOGRAPHIC CHARACTERISTICS**

**Institution**

1. Banaadir university
2. Jamhuriya University of Science and Technology
3. Mogadishu University
4. SIMAD University

**Degree Program**

1. Laboratory Sciences
2. Medicine and Surgery
3. Nursing and Midwifery Sciences
4. Public Health

**Age**

1. 15-25 years
2. 26-30 years
3. 31-40 years
4. More than 40 years

**Gender Male**

1. Female
2. Male

**Marital status**

1. Married
2. Single
3. Widowed/Divorced

**Level of study**

1. Clinical
2. Pre-clinical

**Family size**

1. 0-5
2. 6-10
3. More than10

**SECTION B: EXPOSURE TO RISK FACTORS FOR DEPRESSION**

**Failed an examination?**

1. No
2. Yes

**Emotional problems**

1. No
2. Yes

**Loss of a friend or family member**

1. No
2. Yes

**Financial challenges**

1. No
2. Yes

**Chronic illness/surgery**

1. No
2. Yes

**Physical abuse or trauma**

1. No
2. Yes

**Sexual abuse/violence**

1. No
2. Yes

**Substance abuse or addiction**

1. No
2. Yes

**Unplanned pregnancy**

1. No
2. Yes

**Trouble with school authorities**

1. No
2. Yes

**SECTION C: PATIENT HEALTH QUESTIONNAIRE-9 (PHQ-9).**

**Little interest or pleasure in doing things.**

1. Not at all
2. Several days
3. More than half the days
4. Nearly every day

**Feeling down, depressed, or hopeless.**

1. Not at all
2. Several days
3. More than half the days
4. Nearly every day

**Trouble falling or staying asleep or sleeping too much**

1. Not at all
2. Several days
3. More than half the days
4. Nearly every day

**Feeling tired or having little energy**

1. Not at all
2. Several days
3. More than half the days
4. Nearly every day

**Poor appetite or overeating**

1. Not at all
2. Several days
3. More than half the days
4. Nearly every day

**Feeling bad about yourself or that you are a failure or have let yourself or your family down.**

1. Not at all
2. Several days
3. More than half the days
4. Nearly every day

**Trouble concentrating on things such as reading the newspaper or watching television.**

1. Not at all
2. Several days
3. More than half the days
4. Nearly every day

**Moving or speaking so slowly that other people could have noticed? or the opposite-being so uneasy or restless that you have been moving around a lot more than usual?**

1. Not at all
2. Several days
3. More than half the days
4. Nearly every day

**Thoughts that you would be better off dead or hurting yourself in some way.**

1. Not at all
2. Several days
3. More than half the days
4. Nearly every day
